# Supplementary material for: Dementia Education for Workforce Excellence: Evaluation of a Novel Bichronous Approach
Source: Healthcare (Basel). 2024 Mar 5;12(5):590. doi: 10.3390/healthcare12050590 (PMC10931610; doi:10.3390/healthcare12050590)
Supplement: Supplementary file 1 [file healthcare-12-00590-s001.zip › healthcare-2844806-supplementary.pdf]

**Title of Project: A BLENDED APPROACH TO DEMENTIA TRAINING FOR EXCELLENCE [BADTE]**

**<u>Interview Schedule</u>**

Introductions

Establishing Identity and Verbal Consent

Establishing preferred mode of interview – Audio / Video

1. Before we start, can you please remind us when you participated in the Dementia training?
2. Please tell us what motivated you to participate in the Dementia training?
3. Have you accessed any dementia training prior to this?
4. Please tell us what your role was in the Dementia training programme – Participant / Expert Group Member?
5. What were your expectations of the training before you started?
6. If you could choose 5 words to describe your experience of the training, what would they be?
7. How would you describe your experience with the blended approach to this training?
8. What has changed in your understanding of dementia or the need for dementia training since taking the training for you personally?

9. How has this training informed or influenced your academic / clinical practice?

10. How have you applied what you learned in the training in your everyday life?

11. What would be the top 3 things you took away from your training?

12. What would you have liked to have experienced more in the training?

13. If you could make 1 change to the Dementia training what would it be?

14. Any other suggestions or thoughts.
